# Evaluation of the Antioxidant Activity of Aqueous and Methanol Extracts of *Pleurotus ostreatus* in Different Growth Stages

**DOI:** 10.3389/fmicb.2016.01099

**Published:** 2016-07-12

**Authors:** Ivette González-Palma, Héctor B. Escalona-Buendía, Edith Ponce-Alquicira, Maura Téllez-Téllez, Vijai K. Gupta, Gerardo Díaz-Godínez, Jorge Soriano-Santos

**Affiliations:** ^1^Department of Biotechnology, Metropolitan Autonomous University, Campus IztapalapaMexico, Mexico; ^2^Laboratory of Biotechnology, Research Center for Biological Sciences, Autonomous University of TlaxcalaTlaxcala, Mexico; ^3^Laboratory of Mycology, Biological Research Center, Autonomous University of Morelos StateMorelos, Mexico; ^4^Molecular Glycobiotechnology Group, Discipline of Biochemistry, National University of Ireland GalwayGalway, Ireland

**Keywords:** antioxidant activity, chelating activity, polyphenols compounds, growth stages, *Pleurotus ostreatus*

## Abstract

Total polyphenols and flavonoids contents, as well as ferric reducing antioxidant power (FRAP), metal ions chelating activity, reducing power assay and scavenging activity of DPPH and ABTS radicals in aqueous and methanolic extracts obtained from mycelium, primordium, and fruiting body of *Pleurotus ostreatus* in both fresh as dry, were evaluated. The total polyphenol content of dried samples was higher in aqueous extracts obtained both in room temperature and boiling. The total polyphenol content of the fresh samples obtained at room temperature and boiling was higher in aqueous extract of mycelium and in the methanolic extract of the fruiting body. In general, flavonoids represented a very small percentage of the total polyphenol content. The antioxidant activity measured by the FRAP method of extracts from fresh samples were higher with respect to the dried samples. The results of the metal ion chelating activity indicate that all extracts tested had acted. The reducing power of all samples was concentration dependent. In general, the extracts of dried samples showed higher reducing power than the extracts of fresh samples and tend to show greater reducing power by aqueous than methanolic extracts. It was observed that the DPPH and ABTS radical scavenging activities were positively correlated to the concentration of the extract. The results suggested that antioxidant activity could be due to polyphenols, but mainly by different molecules or substances present in the extracts. Overall, the fruiting body of *P. ostreatus* showed the best results and the possibility of continuing to investigate its functional properties of this fungus is opened. This is the first report where the antioxidant activity of *P. ostreatus* in different growth stage was reported.

## Introduction

Oxidative stress, caused by endogenous factors such as reactive oxygen species (ROS) including the hydroxyl radical, superoxide anion radical, hydrogen peroxide, singlet oxygen, nitric oxide radical, hypochlorite radical, etc., and exogenous factors such as smoking, ionizing radiation, pollution, organic solvents, pesticides, etc., are able to attack nucleic acids, proteins, enzymes, and other small molecules causing loss of structure and function. In the human body, there is a balance between the amount of free radicals produced and antioxidants (Mau et al., 2002; Adebayo et al., 2012). Still, it is necessary to enrich the diet with antioxidants (Adebayo et al., 2012) contained in food to help the organism to reduce oxidative damage (Joan-Hwa et al., 2002; Fatih et al., 2010).

Recently, it has been reported the role in health of natural antioxidants from different herbs and infusions (Chye et al., 2008; Fatih et al., 2010), seeds, oil seeds, cereals, vegetables, fruits, leaves, roots, and spices (Mau et al., 2002). In addition to the phytochemicals, fungi have also been considered as a source of biologically active substances that can be used to reduce oxidative damage in humans and useful in disease prevention (Chye et al., 2008; Fatih et al., 2010; Adebayo et al., 2012; Shirmila and Radhamany, 2012). Fungi produce a considerable amount of metabolites, including vitamin C, vitamin E, and beta-carotene as well as phenolic compounds, known as excellent antioxidants (Barros et al., 2007; Chye et al., 2008; Yin et al., 2008; Fatih et al., 2010; Keles et al., 2011; Adebayo et al., 2012; Shirmila and Radhamany, 2012).

Several research had proven the antioxidant activity of different mushrooms. Gan et al. (2013) quantified total content of polyphenols and flavonoids, as well as, antioxidant activity of the extracts obtained with 60% ethanol and water of *Agaricus bisporus* (white button mushroom) and *A. brasiliensis* (Brazilian mushroom). The aqueous extract of *A. bisporus* had a higher content of polyphenols. Both extracts of *A. brasiliensis* showed higher content of total flavonoids than extracts of *A. bisporus*. Pornariya and Kanok-Orn (2009) investigated the antioxidant properties of aqueous and 95% ethanol extracts of two species of fungi, *Pleurotus ostreatus* and *P. sajor-caju*, obtained from a local farm in Thailand. The aqueous extracts showed the highest amount of total polyphenols and better antioxidant activity than ethanol extracts for both fungi. Jeena et al. (2014) investigated the antioxidant activity and the total polyphenol amount, of methanol extracts of mycelium and fruiting body of *P. sajor-caju, P. ostreatus* and *P. sapidus*. In general, fruiting bodies showed the highest antioxidant activity and reducing power, while the mycelium showed the highest chelating activity.

The bioactivity of polyphenols can be related by their ability to chelate metals, capability of inhibiting or reducing different enzymes such as cyclooxygenase, lipoxygenase and telomerase and free radical scavenging (Yin et al., 2008; Korley et al., 2014). In general, polyphenolic compounds with antioxidant activity are multifunctional and act according to the majority of these mechanisms. Furthermore, it is known that the antioxidant properties depend on the type of solvent used in the extraction and complexity of compounds, since must involve different methods to determine their antioxidant activity (Gioti et al., 2009).

*Pleurotus ostreatus* is a fungus very important because is industrially produced as food (oyster mushroom), a ligninolytic enzyme producer and as bioremediation agents in decontamination processes of materials rich in phenolic compounds, and recently as a biocompounds source (da Luz et al., 2012). During the growth, different metabolites are produced, when the microorganism is in the exponential phase of growth, are produced those called primary metabolites and is known that secondary metabolites start their production by some sort of stress observed by the cells, this stage is when the microorganism not grow anymore but still metabolically active (Pichersky and Gang, 2000). The aim of this study was evaluated antioxidant activity of aqueous and methanolic extracts of *P. ostreatus* at different growth stages of developing on wheat straw.

## Materials and Methods

### Organism and Culture Conditions

*Pleurotus ostreatus* (32783) from the American Type Culture Collection (ATCC) grown on malt extract agar (MEA) at 28°C for 7 days (d) was used. The spawn was obtained by inoculation of wet and sterile wheat grains (500 g) with 10 mycelium plugs taken from the peripheral of a colony by using a sterile cork borer (4 mm diam). Spawn of *P. ostreatus* (500 g) was inoculated on pasteurized wheat straw (5 kg at 70% of humidity), the mix was collocated inside of a plastic bag, and incubated in a dark room at 25°C for 15 days, after that, was exposed to daylight at 21°C for 8 days. During this process, mycelium grown on wheat straw, primordia and fruiting bodies were obtained.

### Extracts

For obtaining extracts, mycelium, primordia, and fruiting bodies, both fresh and dried samples were used. Samples were dried in an oven at 58°C for 24 h. Aqueous and methanolic extracts in both room temperature and boiling were obtained. For extracts obtained by boiling, the Soxhlet apparatus was used for methanolic extracts (Association of Official Analytical Chemists [AOAC], 2000) and for the aqueous extracts were shaken in boiling for 5 min. In all cases, 0.5 g of sample in a final volume of 10 mL, was shaken for 30 min (Miliauskas et al., 2004). In the case of aqueous extracts, the supernatant was recovered by centrifugation and for methanolic extraction, a rotary evaporator was used until the total recovery of the solvent and the residue was solubilized in 5% dimethylsulfoxide. The results were reported in dry basis, considering 90% moisture in the fresh samples.

### Total Phenolic Content Assay

The total phenolic content was measured according to the method of Singleton et al. (1999). Slowly, 0.5 mL of sample was added to 4.5 mL of distilled water and was mixed with 0.2 mL of the Folin–Ciocalteu phenol reagent and 0.5 mL saturated solution of Na_2_CO_3_, finally 4.3 mL of distilled water was added to the solution. The reaction mixtures were incubated for 60 min in the dark at room temperature and then, the absorbances were measured at 725 nm. Total phenolic content was expressed as mg of Gallic acid equivalents (GAE) per gram of dry sample (mg GAE/g).

### Flavonoid Content

The flavonoid content was determined by the colorimetric method of aluminum chloride according to methodology previously described by Chia-Chi et al. (2002). Slowly, 0.5 mL sample was taken and was mixed with 1.5 mL of 95% ethanol, 0.1 mL of 10% aluminum chloride, 0.1 mL of 1 M potassium acetate and 2.8 mL of distilled water. This mixture was incubated at room temperature for 30 min in darkness. Finally, absorbance at 415 nm was read. The flavonoid content was calculated in mg quercetin equivalents per g of dried sample (mg QE/g).

### Ferric Reducing Antioxidant Power (FRAP)

Ferric reducing antioxidant power assay was measured according to the procedure described by Sudha et al. (2012) with some modifications. The FRAP reagent contained 2.5 mL of a 10 mM TPTZ (2,4,6-Tripyridyl-s-Triazine) solution in 40 mM HCl, 2.5 mL of 10 mM FeCl_3_∙6H_2_O, and 25 mL of 300 mM acetate buffer (pH 3.6). It was freshly prepared and warmed at 37°C. A 900 μL FRAP reagent was mixed with 90 μL water and 30 μL of the extract. The reaction mixture was incubated at 37°C for 10 min and the absorbance was measured at 593 nm. FRAP was expressed as μM de FeSO_4_/g of dry sample.

### Metal Ion Chelating Activity

Metal ion chelating activity was measured according to the method described by Zhengjun et al. (2008). Slowly, 1 mL of sample solution was premixed with 0.05 mL of FeCl_2_ solution (2 mM) and 1.85 mL of double distilled water. Afterward, 0.1 mL of ferrozine solution (5 mM) was added and mixed vigorously. The absorbance was determined at 562 nm after the mixture stood for 10 min at the room temperature. Double distilled water was used as a control. The chelating effect was calculated by using the following equation.

$$\%\mathrm{chelating}\;\mathrm{effect}\;=\;[(A_{\mathrm{control}}\;-\;A_{\mathrm{sample}}){/A}_{\mathrm{control}}]\;\times \;100$$

A_control_ = Absorbance of negative control at the moment of solution preparation

A_sample_ = Absorbance of sample after

The EC_50_ values were calculated from the graph which represents the concentration of the sample required to scavenge 50% of the chelating effect. The EC_50_ is often used to express the amount or concentration of extracts needed to scavenge 50% of the free radicals. Metal ion chelating activity was expressed as mg GAE/L.

### Reducing Power Assay

The method was based on Hue et al. (2012) procedures with modifications. The extracts (0.050 mL) of the different concentrations were added with 0.2 mL of 0.2 M phosphate buffer (pH 6.6) and 0.2 mL of 1% potassium ferricyanide. The mixture was incubated in the water bath for 20 min at 50°C. Trichloroacetic acid (0.25 mL) was added to the mixture and was centrifuged at 1000 rpm for 10 min at room temperature. The supernatant (0.5 mL) was added with 0.5 mL of deionised water and 0.1 mL of 0.1% FeCl_3_. Absorbance was recorded at 700 nm. Reducing power assay was expressed as mg GAE/L.

### Scavenging Activity of DPPH Radical

DPPH radical scavenging activity was determined according to Moraes-de-Souza et al. (2008) with some modifications. The reaction mixture consisted of 0.5 mL of extract, 3 mL of methanol, and 0.3 mL of 0.5 mM 2,2-diphenyl-1-picrylhydrazyl (DPPH) radical solution in methanol. After incubation for 45 min, absorbance was determined in a spectrophotometer at 517 nm. The antioxidant activity was calculated by using the following equation.

$$\%\mathrm{inhibition}\;=\;[(A_{\mathrm{control}}\;-\;A_{\mathrm{sample}}){/A}_{\mathrm{control}}]\;\times \;100$$

A_control_ = Absorbance of negative control at the moment of solution preparation

A_sample_ = Absorbance of sample after 45 min

### Scavenging Activity of ABTS Radical

ABTS radical scavenging activity was determined according to Re et al. (1999) with some modifications. 2,2′-azino-bis(3-ethylbenzothiazoline-6-sulphonic acid) (ABTS) was dissolved in water to a 7 mM concentration. ABTS radical cation (ABTS^∙+^) was produced by reacting ABTS stock solution with 2.45 mM potassium persulfate (final concentration) and allowing the mixture to stand in the dark at room temperature for 12–16 h before use. The ABTS^∙+^ solution was diluted with water to an absorbance of 0.70 (±0.02) at 734 nm. The reaction mixture consisted of 0.07 mL of extract and 3 mL of the ABTS radical. After incubation for 6 min, absorbance was determined in spectrophotometer at 734 nm. The antioxidant activity was calculated by using the following equation.

$$\%\mathrm{inhibition}\;=\;[(A_{\mathrm{control}}\;-\;A_{\mathrm{sample}}){/A}_{\mathrm{control}}]\;\times \;100$$

A_control_ = Absorbance of negative control at the moment of solution preparation

A_sample_ = Absorbance of sample after 6 min

The EC_50_ values were calculated from the graph which represents the concentration of the sample required to scavenge 50% of the ABTS or DPPH free radicals. The EC_50_ is often used to express the amount or concentration of extracts needed to scavenge 50% of the free radicals. ABTS and DPPH were expressed as mg GAE/L.

### GC Mass Analysis

The extracts were quantified using gas chromatograph (Agilent Technologies, 7890A GC System) equipped with a column Agilent DB-WAX (30 m × 320 μm × 0.25 μm) coupled to a mass spectrometer Agilent Technologies (5975C V2-MSD with Triple-Axis Detector) with Autosampler and injector (G4513A), programmed at temperature 60°C for 5 min, then 11°C/min to 250°C. The injector flow rate was 250°C; carrier gas was He of 99.9995% purity, column flow rate 1.2926 mL/min.

### Statistical Analysis

All the analyses were run in triplicate and averaged. The data were analyzed using Sigma Stat software. The antioxidant values for the extracts were evaluated with the one-way ANOVA and Tukey’s. *P* values less than 0.050 were considered to be statistically significant.

## Results

### Polyphenols Content

The polyphenols content depends on the type of sample and solvent and temperature used for extraction. In general, extracts of the fruiting body had a high amount of polyphenols, followed by primordium and by the mycelium (**Table 1**). In the case of aqueous extracts of the fruiting body and primordium both dried, obtained at room temperature were observed 11.36 ± 0.04 and 8.94 ± 0.03 mg GAE/g, respectively. For aqueous extracts obtained by boiling showed very similar values of polyphenols content (around 4 mg GAE/g) and in these samples, the smallest value was reported for mycelium extract obtained at room temperature. The methanol extracts of dried samples showed low values (between 0.62 ± 0.01–2.39 ± 0.02 mg GAE/g). For fresh samples, methanolic extract of primordium and aqueous extract of the fruiting body, showed the highest polyphenols values, 12.06 ± 0.02 and 9.92 ± 0.05 mg GAE/g, respectively. The aqueous extracts showed similar values about 4–5 mg GAE/g. Song and Leo (2008) quantified polyphenols in aqueous extracts obtained with hot water of *Ganoderma lucidum*, reported 0.128–2.78 mg GAE/mL, and for *A. brasiliensis* 0.512 and 0.0853 mg GAE/mL. The values reported in previous works were lower than those obtained in this research, with the same treatment and reported in the same units. Yim et al. (2009) reported in aqueous extracts of *Pleurotus* sp. and *P. ostreatus* 9.01 and 7.23 mg GAE/g, respectively. Both values are lower than those reported in this study for the aqueous extract of fresh fruiting body obtained by boiling (9.92 ± 0.05 mg GAE/g) and the aqueous extract of the dry fruiting body obtained at room temperature (11.36 mg GAE/g). In methanol extracts of *Pleurotus* sp. (5.06 mg GAE/g) and *P. ostreatus* (6.03 mg GAE/g), the total polyphenols values were lower than those observed in this research in methanol extracts of fresh primordium and fresh fruit body, both prepared by boiling (12.06 ± 0.02 and 7.1 ± 0.02 mg GAE/g, respectively). Furthermore, Nuran et al. (2012) reported 32.21 mg GAE/g in the methanol extract of *P. eryngii*.

**Table 1:** Total polyphenol content of *Pleurotus ostreatus.*

	Dry				Fresh			
	Aqueous extracts		Methanolic extracts		Aqueous extracts		Methanolic extracts	
	T1	T2	T1	T2	T1	T2	T1	T2
Micelium	1.65 ± 0.03^a^	4.09 ± 0.23^a^	0.99 ± 0.01^a^	0.62 ± 0.01^a^	5.09 ± 0.01^a^	8.64 ± 0.01^a^	2.1 ± 0.01^a^	1.52 ± 0^a^
Primordium	8.94 ± 0.03^b^	4.22 ± 0.20^a^	1.42 ± 0.03^b^	1.24 ± 0.02^b^	4.16 ± 0.02^b^	4.21 ± 0.01^b^	6.8 ± 0.02^b^	12.06 ± 0.02^b^
Fruiting body	11.36 ± 0.04^c^	4.65 ± 0.26^a^	2.39 ± 0.02^c^	1.58 ± 0.04^c^	5.05 ± 0^c^	9.92 ± 0.05^c^	4.9 ± 0.02^c^	7.1 ± 0.02^c^

*Values are the average of three replicates ± DS and expressed in mg GAE/g dry basis. Means followed by different letters are significantly different (*p* ≤ 0.05). T1 = extract obtained at room temperature, T2 = extract obtained by boiling.*

### Quantification of Flavonoids

The content of flavonoids found in all samples was low, the values were in the range of 0.011 ± 0.001 to 1.04 ± 0.008 mg QE/g, and specifically the aqueous extracts of fresh fruiting body and fresh primordium reported zero (**Table 2**). Flavonoids represented a very small percentage of the total polyphenol content. Arbaayah and Umi (2013) reported a high content of flavonoids from ethanol extracts of various species of *Pleurotus*, with values of 1.40 to 29.80 mg QE/g. Jan-Ying et al. (2011) reported the flavonoids content from ethanolic, cold water and hot water extracts of two strains of *Grifola frondosa*; values were 1.09–3.05 and 0.11–0.76 mg EQ/g of sample dry, respectively. Sudha et al. (2012) reported the flavonoids content in ethyl acetate, methanol and hot water extracts of *P. eous*, reaching values of 6.38 to 7.79 mg catechin equivalents (CAE)/g extract. However, Gil-Ramírez et al. (2016), reported that the mushrooms do not contain flavonoids, and those found in the hyphae could be due to the facility of these organisms to absorb many nutrients and compounds from the substrate where they grow or from neighboring plants by spreading their hyphae or forming mycorrhizae. Some plants release certain flavonoids for regulating the symbiotic plant–microbe interactions defining the species tolerated to grow on their roots. Flavonoids are reported as antifungal compounds, because plants produce them as protection against fungal infections, then, these compounds might negatively affect fungal growth.

**Table 2:** Total flavonoids content of *P. ostreatus.*

	Dry				Fresh			
	Aqueous extracts		Methanolic extracts		Aqueous extracts		Methanolic extracts	
	T1	T2	T1	T2	T1	T2	T1	T2
Micelium	0.120 ± 0.01^a^	0.192 ± 0.01^a^	0.026 ± 0.002^a^	0.011 ± 0.001^a^	1.04 ± 0.008^a^	0.140 ± 0.01^a^	0.055 ± 0.01^a^	0.077 ± 0.001^a^
Primordium	0.068 ± 0.001^b^	0.143 ± 0.01^b^	0.025 ± 0.001^a^	0.020 ± 0.003^b^	0^b^	0.085 ± 0.01^b^	0.200 ± 0.003^b^	0.734 ± 0.002^b^
Fruiting body	0.069 ± 0.003^b,c^	0.131 ± 0.01^c^	0.049 ± 0.004^b^	0.024 ± 0.002^b,c^	0^b,c^	0.100 ± 0.05^c^	0.110 ± 0.001^c^	0.260 ± 0.002^c^

*Values are the average of three replicates ± DS and expressed in mg QE/g dry basis. Means followed by different letters are significantly different (*p* ≤ 0.05). T1 = extract obtained at room temperature, T2 = extract obtained by boiling.*

### Ferric Reducing Antioxidant Power (FRAP)

The antioxidant activity measured by FRAP method was observed in all extracts (**Table 3**). In general, values of antioxidant activity of extracts of fresh samples were higher with respect to the dried samples. The highest values were 166.5 ± 0.10 and 113.9 ± 0.24 μM de FeSO_4_/g in methanolic extracts of fresh primordium and in aqueous extracts of fresh fruiting body, respectively, both obtained by boiling. It was observed that the antioxidant activity measured by FRAP was positively correlated to the concentration of the polyphenols. Only, the aqueous extracts of dry samples were not positively correlated to the concentration of the polyphenols. The values observed with this method were generally low compared with those reported in other studies, Keles et al. (2011) found in methanol extracts of *P. ostreatus* and *P. dryinus* high antioxidant 11600 and 2385.71 μM de FeSO_4_/g. They also found in their methanol extracts a low antioxidant activity of mushroom *Hydnum repandum* with 145.50 μM de FeSO_4_/g.

**Table 3:** Ferric reducing antioxidant power (FRAP) of *P. ostreatus.*

	Dry				Fresh			
	Aqueous extracts		Methanolic extracts		Aqueous extracts		Methanolic extracts	
	T1	T2	T1	T2	T1	T2	T1	T2
Micelium	14.79 ± 1.6^a^	9.47 ± 1.6^a^	4.22 ± 0.80^a^	3.56 ± 0.13^a^	23 ± 0.12^a^	66.4 ± 0.69^a^	14.8 ± 0.01^a^	9.29 ± 0.04^a^
Primordium	6.86 ± 0.38^b^	17.6 ± 1.0^b^	8.97 ± 0.25^b^	6.52 ± 0.58^b^	4.87 ± 0.05^b^	22.7 ± 0.19^b^	45.7 ± 0.15^b^	166.5 ± 0.10^b^
Fruiting body	9.7 ± 0.57^c^	33 ± 0.20^c^	24.53 ± 1.2^c^	11.95 ± 0.23^c^	19.7 ± 0.15^c^	113.9 ± 0.24^c^	66.9 ± 0.34^c^	65.3 ± 0.42^c^

*Values are the average of three replicates ± DS and expressed in mg μM de FeSO_*4*_/g dry basis. Means followed by different letters are significantly different (*p* ≤ 0.05). T1 = extract obtained at room temperature, T2 = extract obtained by boiling.*

### Metal Ion Chelating Activity

The results of the metal ion chelating activity indicate that all extracts tested had activity (**Table 4**). The extract that showed the highest chelating activity was the methanol extract of dried mycelium obtained by boiling (EC_50_ = 13.17 ± 0.13 mg GAE/L), followed by the aqueous extract of the dried fruiting body obtained by boiling, with an EC_50_ = 25.53 ± 0.80 mg GAE/L. Although all extracts showed chelating activity, some of them had higher values, for example EC_50_ = 1932.06 ± 0.95 mg GAE/L for the methanol extract obtained by boiling of dried primordium. Chye et al. (2008) reported chelating activity in petroleum ether and methanol extracts (obtained at room temperature) of the mushrooms *Pleurotus* sp. and *P. florida.* The petroleum ether extracts of *Pleurotus* sp. and *P. florida* showed chelating activity with EC_50_ = 16.26 and 400 mg GAE/mL and the methanolic extracts with EC_50_ = 2.01 and 260 mg GAE/mL, respectively. The methanolic extract (obtained by boiling) of the dried micelium of this study, showed better chelating activity (EC_50_ = 13.17 ± 0.13 mg GAE/L) than the petroleum ether extract of *Pleurotus* sp. The petroleum ether and methanolic extract of *P. florida* presented higher EC_50_ values compared with the aqueous extract (obtained by boiling) of dried and fresh fruiting bodies reported in this research (EC_50_ = 25.53 ± 0.80 and 42.4 ± 0.72 mg GAE/L, respectively). Nuran et al. (2012) reported high chelating activity of methanol extract of the mushroom *P. eryngii* collected from different regions of the Tunceli province of Turkey (EC_50_ = 470.23, 218.31, and 292.99 mg GAE/mL for Ovacik, Pulumur and City center, respectively). Comparing the previous results with those reported in our study, the value obtained from first region was higher than the reported for aqueous extract (obtained at room temperature and boiling) of fresh fruiting body, as well as of the methanolic extract (obtained at room temperature) of fresh fruiting body and the aqueous extract (obtained by boiling) of the dried fruiting body. The value obtained by second and third region was higher than the value reported for the aqueous extracts (obtained by boiling) of dried and fresh fruiting bodies and methanolic extract (obtained at room temperature) of fresh fruiting body.

**Table 4:** Metal ions chelating activity of *P. ostreatus.*

	Dry				Fresh			
	Aqueous extracts		Methanolic extracts		Aqueous extracts		Methanolic extracts	
	T1	T2	T1	T2	T1	T2	T1	T2
Micelium	54.1 ± 1.10^a^	71.79 ± 1.05^a^	479.02 ± 0.95^a^	13.17 ± 0.13^a^	167.7 ± 0.64^a^	389.9 ± 0.61^a^	361 ± 0.37^a^	344.2 ± 0.74^a^
Primordium	192.43 ± 1.25^b^	51.76 ± 1.20^b^	327.96 ± 0.59^b^	1932.06 ± 0.95^b^	300.8 ± 0.60^b^	205.2 ± 0.72^b^	241.8 ± 0.50^b^	399.7 ± 1.63^b^
Fruiting body	485.27 ± 0.63^c^	25.53 ± 0.80^c^	544.2 ± 0.23^c^	680.37 ± 0.6^c^	412.6 ± 0.95^c^	42.4 ± 0.72^c^	171.4 ± 0.70^c^	501.8 ± 0.78^c^

*Values are the average of three replicates ± DS and expressed in EC_*50*_ in mg GAE/L dry basis. Means followed by different letters are significantly different (*p* ≤ 0.05). T1 = extract obtained at room temperature, T2 = extract obtained by boiling.*

### Reducing Power Assay

**FIGURE 1:**
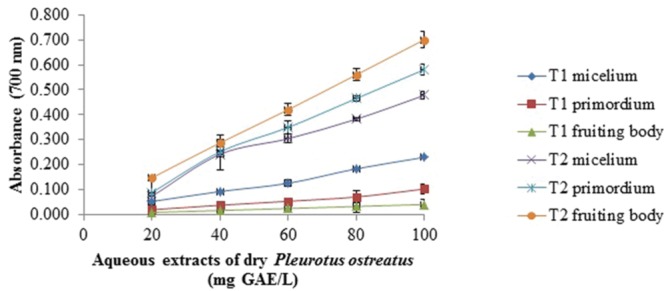
**Reducing power assay of aqueous extracts of dry *Pleurotus ostreatus*.** T1 = extract obtained at room temperature, T2 = extract obtained by boiling. Values are the average of three replicates ± DS.

**FIGURE 2:**
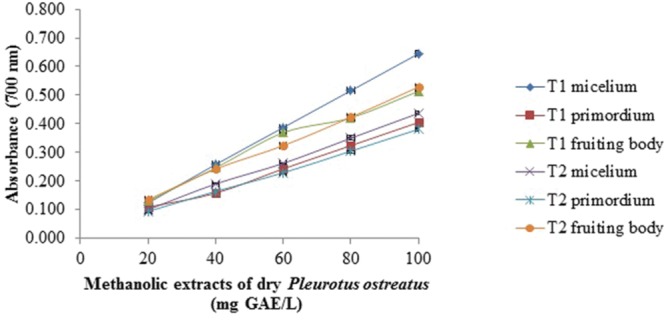
**Reducing power assay of methanolic extracts of dry *P. ostreatus.*** T1 and T2 as **Figure 1** Values are the average of three replicates ± DS.

**FIGURE 3:**
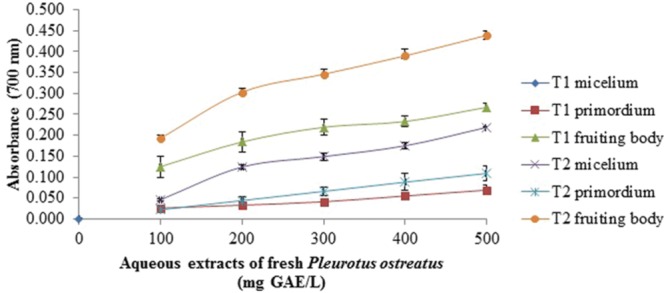
**Reducing power assay of aqueous extracts of fresh *P. ostreatus.*** T1 and T2 as **Figure 1** Values are the average of three replicates ± DS.

**FIGURE 4:**
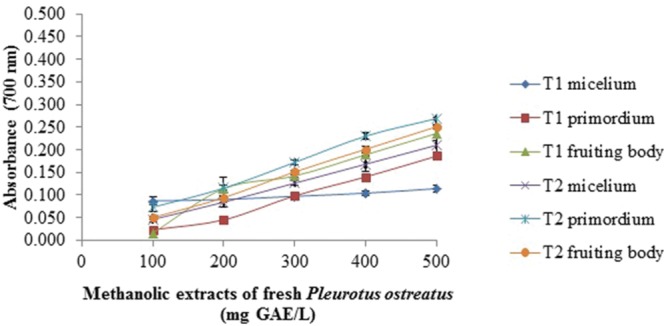
**Reducing power assay of methanolic extracts of fresh *P. ostreatus*.** T1 and T2 as **Figure 1** Values are the average of three replicates ± DS.

The reducing power of all samples was concentration dependent (**Figures 1–4**). In general, the extracts of dried samples showed higher reducing power than the extracts of fresh samples and tend to show greater reducing power by aqueous than methanolic extracts. In the case of extracts from dried samples, the highest value of reducing power it showed by aqueous extract obtained by boiling of fruiting body with a value of 0.701 ± 0.003, and the methanol extract obtained at room temperature of mycelium showed 0.645 ± 0.009, both at a concentration of 100 mg GAE/L (**Figures 1** and **2**). For extracts of fresh samples, the highest values were obtained in water extracts of fruiting body by boiling with a value of 0.439 ± 0.011 (**Figure 3**) and methanolic extracts of the fruiting body and primordium both by boiling, with values of 0.269 ± 0.003 and 0.251 ± 0.005, respectively (**Figure 4**), both at a concentration of 500 mg GAE/L. Arbaayah and Umi (2013) reported reduced power of ethanolic extracts of mushrooms of *Pleurotus* genus and *Schizophyllum commun.* The higher reducing power (at 10 mg sample/mL) was shown for *P. djamor* var. *djamor* with an absorbance of 0.874, followed by *P. djamor* var. *roseus* (0.771), *Schizophyllum commune* (0.568), *P. pulmonarius* (0.429), and finally *P. ostreatus* (0.397). Shimada et al. (1992) and Arbaayah and Umi (2013) reported that the reducing power of the mushroom extracts can be due to the ability of hydrogens donation that stabilize the molecules by acceptance of hydrogen ions in the extracts. The properties of the reducing power can be an indicator of antioxidant potential of compound evaluated (Meir et al., 1995).

### Scavenging Activity of DPPH Radical

It was observed that the DPPH radical scavenging activity was positively correlated to the concentration of the extract. EC_50_ values of the extracts evaluated in this study are shown in **Table 5** In general, extracts of dried samples showed higher DPPH radical scavenging activity. It was observed that aqueous extracts showed generally higher DPPH radical scavenging activity than methanol extract samples in both dry and fresh. Furthermore, treatment of boiling for extraction favored the DPPH radical scavenging activity, since these extracts showed higher activity than the extracts obtained at room temperature, except for the methanol extract of dry fruiting body and dry primordium obtained at room temperature, which they showed the lowest values of EC_50_ (30.89 ± 1.83 and 26.99 ± 0.47 mg GAE/L, respectively). Keles et al. (2011) found a DPPH radical scavenging activity, of methanol extract of *A. bisporus, P. dryinus, Boletus edulis*, and *P. ostreatus* with EC_50_ = 78.43, 58.06, 38.31, and 29.66 mg GAE/mL, respectively (EC_50_ calculated from the original values reported by the authors). The values of DPPH radical scavenging activity were higher compared with the obtained in this investigation in the methanolic extract (obtained at room temperature) of the dried fruiting body (EC_50_ = 26.99 ± 0.47 mg GAE/L). Shirmila and Radhamany (2013) reported DPPH radical scavenging activity of methanol extract of *Macrolepiota mastoidea* (EC_50_ = 20.35 mg GAE/mL), obtained by apparatus Soxhlet (EC_50_ calculated from the values reported by the authors).

**Table 5:** DPPH radical scavenging activity of *P. ostreatus.*

	Dry				Fresh			
	Aqueous extracts		Methanolic extract		Aqueous extracts		Methanolic extract	
	T1	T2	T1	T2	T1	T2	T1	T2
Micelium	53.57 ± 1.26^a^	185.41 ± 0.80^a^	47.35 ± 1.03^a^	36.18 ± 1.48^a^	10808 ± 0.160^a^	389.3 ± 1.19^a^	283.7 ± 0.80^a^	189.7 ± 1.11^a^
Primordium	523.17 ± 0.75^b^	43.32 ± 0.94^b^	30.89 ± 1.83^b^	45.64 ± 1.16^b^	685.4 ± 1.05^b^	474 ± 1.59^b^	241.2 ± 0.95^b^	291.7 ± 0.35^b^
Fruiting body	775.93 ± 1.28^c^	36.86 ± 1.84^c^	26.99 ± 0.47^c^	66 ± 0.24^c^	436.8 ± 0.16^c^	244.4 ± 0.70^c^	264.7 ± 1.23^c^	185.3 ± 0.99^c^

*Values are the average of three replicates ± DS and expressed in EC_*50*_ in mg GAE/L dry basis. Means followed by different letters are significantly different (*p* ≤ 0.05). T1 = extract obtained at room temperature, T2 = extract obtained by boiling.*

### Scavenging Activity of ABTS Radical

ABTS radical scavenging activity was positively correlated to the concentration of the extract. In this analysis, the dried samples showed higher radical scavenging activity of ABTS than the fresh samples (**Table 6**). In the fresh samples it was observed that in both aqueous and methanolic extracts, the highest activity was observed in the mycelium followed by the primordium and at the end by the fruiting body. In general, the dried samples had higher activity in the methanolic extract. The methanol extract of the dried primordium obtained at room temperature showed the highest activity with an EC_50_ = 22.89 ± 0.37 mg GAE/L. Shirmila and Radhamany (2013) reported ABTS radical scavenging activity, of methanol extract of *Macrolepiota mastoidea* with EC_50_ = 9.9 mg GAE/mL (EC_50_ calculated from the original value reported by the authors). The value reported was lower compared with that observed in this investigation in methanolic extract obtained at room temperature and boiling of the dried fruiting body with EC_50_ = 217.24 ± 1.31 and 76.63 ± 0.070 mg GAE/L, respectively. Guzmán et al. (2009) reported radical scavenging activity of methanol extract of dried *P. djamor* (EC_50_ of 0.0293 mg sample/mL). The methanolic extract obtained at room temperature had more radical scavenging activity than the methanolic extract obtaining by boiling of the dried fruiting body obtained in this investigation with EC_50_ de 32.26 and 136.98 mg sample/mL, respectively.

**Table 6:** ABTS radical scavenging activity of *P. ostreatus.*

	Dry				Fresh			
	Aqueous extracts		Methanolic extract		Aqueous extracts		Methanolic extract	
	T1	T2	T1	T2	T1	T2	T1	T2
Micelium	88.12 ± 0.41^a^	240.88 ± 0.85^a^	86.38 ± 0.64^a^	70.13 ± 0.32^a^	2109 ± 0.08^a^	1225.3 ± 1.52^a^	1167.2 ± 0.52^a^	1023.4 ± 0.14^a^
Primordium	375.71 ± 0.76^b^	97.38 ± 1.38^b^	22.89 ± 0.37^b^	73.78 ± 0.70^b^	653.5 ± 0.33^b^	817.6 ± 0.95^b^	230.3 ± 1.63^b^	880.8 ± 0.30^b^
Fruiting body	317.09 ± 0.85^c^	99.95 ± 0.82^b,c^	76.63 ± 0.070^c^	217.24 ± 1.31^c^	819.3 ± 1.53^c^	532.5 ± 0.84^c^	549.3 ± 0.67^c^	576.3 ± 0.58^c^

*Values are the average of three replicates ± DS and expressed in EC_*50*_ in mg GAE/L dry basis. Means followed by different letters are significantly different (*p* ≤ 0.05). T1 = extract obtained at room temperature, T2 = extract obtained by boiling.*

### GC Mass Analysis

The GC Mass analysis revealed in some samples, mainly of the fruiting body the presence of methyltartronic acid (RT 20.185) which could be the responsiblity of the antioxidant activity. The synonyms of methyltartronic acid are malic acid, isomalic acid, 2,2-dihydroxy-3-oxobutanoic acid, 2-metiltartronic acid, 2 hidroxy-2-methylpropanedioic acid, 2 hydroxy-2-methylmalonate, hydroxymethylpropanedioic acid. On the other hand, in different mushrooms like *Amanita caesarea, Boletus edulis, Gyroporus castaneus, Lactarius deliciosus, Suillus collinitus*, and *Xerocomus chrysenteron* have been found a profile composed of at least five organic acids: citric, ketoglutaric, malic, succinic, and fumaric acids. In addition, those organic acids may have a protective role against various diseases due to their antioxidant activity (Valentao et al., 2005). Petrović et al. (2015) reported the profile of organic acids of the mushroom *Agrocybe aegerita* (Brig.). Malic acid was the most abundant organic acid (1.82 g/100 g), followed by citric acid (0.88 g/100 g), then fumaric acid (0.26 g/100 g) and oxalic acid (0.09 g/100 g).

## Conclusion

All water and methanolic extracts possess phenolics compounds and flavonoids. In the case of the phenolics compounds, the best extracts were the methanolic of fresh primordium obtained by boiling and aqueous of the dry fruiting body obtained at room temperature. In general, the flavonoids content was reported with a low value, representing a very small percentage of the total polyphenol content, however, the majority of the extracts presented antioxidant activity. The best extract in ferric reducing antioxidant power (FRAP) was the methanolic of fresh primordium obtained by boiling. For metal ion chelating activity, the best extract was methanolic of dry mycelium obtained by boiling. The best extract with the reducing power assay was aqueous extract of dry fruiting body obtained by boiling. Methanolic extract obtained at room temperature of the fruiting body and primordium, both dried showed the higher radical scavenging activity of DPPH and ABTS. The different results also suggested that antioxidant activity couldn’t be by polyphenols. The antioxidant activity may be as a result of the presence of different molecules or substances no determined in this study which are present in the extracts. Finally, in this investigation has not found a pattern of behavior at different stages of development of *P. ostreatus*, so the possibility to further investigate the functional properties of this fungus is opened.

## Author Contributions

This work was carried out in collaboration between all authors. Authors JS-S and GD-G designed the study, contributed reagents/materials, and supervised the work in all its aspects. Author IG-P carried out trials and prepared the protocol. Author MT-T managed the literature searches. Author HE-B analyzed the statistical analysis. Authors EP-A and VG followed and supervised this study in all its aspects.

## Conflict of Interest Statement

The authors declare that the research was conducted in the absence of any commercial or financial relationships that could be construed as a potential conflict of interest.
